# OSDI Questions on Daily Life Activities Allow to Detect Subclinical Dry Eye in Young Contact Lens Users

**DOI:** 10.3390/jcm11092626

**Published:** 2022-05-06

**Authors:** José Ángel Pastor-Zaplana, Fernando Borrás, Juana Gallar, M. Carmen Acosta

**Affiliations:** 1 Instituto de Neurociencias, Universidad Miguel Hernández-CSIC, 03550 Sant Joan d’Alacant, Spain; jose.pastorz@umh.es (J.Á.P.-Z.); juana.gallar@umh.es (J.G.); 2 Departamento de Patología y Cirugía, Universidad Miguel Hernández de Elche, 03550 Sant Joan d’Alacant, Spain; 3 Departamento de Estadística, Matemáticas e Informática, Universidad Miguel Hernández de Elche, 03550 Sant Joan d’Alacant, Spain; f.borras@umh.es; 4 Instituto de Investigación Biomédica y Sanitaria de Alicante, 03010 Alicante, Spain

**Keywords:** dry eye disease symptoms, contact lens, OSDI, ocular symptoms evaluation, daily life activities

## Abstract

Dry eye disease (DED) is difficult to detect in young contact lens (CL) wearers, who usually have no signs, mild symptoms and an ocular surface disease index (OSDI) below the DED diagnosis values (OSDI ≥ 13). We investigate if some of the 12 OSDI questions (OSDI A—ocular symptoms; OSDI B—vision-related functionality; OSDI C—environmental triggers) contribute the most to classify young CL as symptomatic. TBUT and tear volume are also measured. Age, gender and refraction error-matched eye glasses (EG) wearers participated as the control. CL and EG data were compared with *t*-test and *z*-test. Confusion matrices and logistic correlation analyses were performed to define the contribution of each OSDI question to classify symptomatic subjects. OSDI classified symptomatic CL better than the tear volume or TBUT values. In CL, only OSDI B and C values were significantly higher in symptomatic vs. asymptomatic subjects (*p* < 0.001), while values of all twelve OSDI questions were significantly higher in symptomatic vs. asymptomatic EG (*p* < 0.05–0.001). All OSDI questions contribute equally to identify symptomatic EG, while only OSDI B questions on daily life visual functions are significant to classify symptomatic CL wearers at risk to develop DED or at a subclinical stage. CL wearers scoring ≥ 2 on the OSDI B questions should be considered for preventive treatments, even if their clinical sings are scarce or absent.

## 1. Introduction

In order to detect ocular discomfort and dryness symptoms, many different questionnaires have been developed, including the ocular surface disease index (OSDI) [1], the five-item dry eye questionnaire (DEQ-5) [2], the dry eye-related quality of life score (DEQS) [3], the impact of dry eye on everyday life (IDEEL) [4], the McMonnies’ questionnaire (MQ) [5], the ocular comfort index (OCI and OCI-I) [6], the symptom assessment in dry eye (SANDE) [7] or the standard patient evaluation of eye dryness (SPEED) [8]. On many occasions, there is no clear correlation between the clinical signs (tear volume and tear stability measurements, cornea %l staining, etc.) and the results of the questionnaires [9,10,11,12,13]. Although many different explanations have been proposed for this issue, one of the simplest is that the questionnaires may detect early symptoms in subjects without clear clinical signs [13], especially in young subjects.

Approximately 140 million people use contact lenses (CL) all over the world [14], and despite the improvement in the composition of materials, design and care systems [14,15,16], a high percentage of CL users suffers adverse effects, such as corneal infections, inflammation, lesions and unpleasant sensations [15,17,18,19]. Among these, dryness and discomfort are the most prevalent [20,21] and the main reason to abandon CL wearing [22]. There is a direct relationship between the time of CL use and the development of ocular discomfort, increasing its severity at the end of the day [23,24,25] and affecting daily life activities. It is not surprising then that the use of CL is one of the risk factors to developing dry eye disease (DED) [26,27], and that specific questionnaires for CL users have been developed, such as the contact lens dry eye questionnaire (CLDEQ) [28], the eight-item contact lens dry eye questionnaire (CLDEQ-8) [29], the contact lens impact on quality of life [30] and the recently designed contact lens discomfort index (CLDI) [31].

Among the plethora of existing questionnaires, OSDI is still the strongest questionnaire [27], used widely to determine ocular discomfort and dryness in both the clinic and research. One of its strengths is that OSDI not only measures symptoms and their frequency, but also environmental triggers and the vision-related impact on the quality of life [27,32,33,34,35,36]. These items are important, as they can bring to light hidden symptoms, leading to the detection of ocular surface diseases in early stages. However, more simple questionnaires, which do not measure daily life activities and quality of life are also widely used in individuals with different conditions, such as in DED patients or CL wearers, an example of such a questionnaire being the subjective evaluation of symptoms of dryness (SESoD), which only measures the intensity of dryness throughout the day [25,37]. Some authors have even designed ad hoc questionnaires that fulfil the necessities of a particular research work, adapting the questionnaire to the characteristics of the group or groups of subjects to be studied. In these ad hoc questionnaires, the researchers select specific questions depending on the interest of the investigation [38]. A drawback of these questionnaires is that they can be biased, so they should be compared with validated questionnaires.

OSDI has twelve questions grouped in three categories or subscales (ocular discomfort, vision-related functionality and environmental factors). A subject is classified as symptomatic when the total OSDI score is equal or over thirteen points [1,27], where all twelve items have the same weight. The aim of this study is to study whether any of the OSDI categories of questions are more relevant when defining symptomatic EG and CL subjects since, to the best of our knowledge, it has not been previously studied.

## 2. Materials and Methods

### 2.1. Subjects

A total of 134 students and staff of the Universidad Miguel Hernández (Spain) participated voluntarily in this observational study performed in young healthy subjects. The inclusion criteria were: EG or soft CL wearing for more than 1 year; age between 18 and 40 years; both sexes; absence of any ocular surface disease. The exclusion criteria were: previous history of corneal or ocular disease: major systemic disease; pregnancy; previous CL wearing in the case of EG participants. EG wearers with similar refraction errors were included as the control group. If a group without refractive errors had been included, it would not have been possible to discern whether the OSDI alterations just reflected the difficulties of CL wearers to perform vision-related activities due to their vision deficit. Young CL and EG volunteers (age range: 18–39 years) of both sexes (103 women, 31 men) and similar refractive errors (−0.5 to −15.5, −0.25 to −1.8 and +0.5 to +1.75 diopters for myopia, astigmatism and hyperopia, respectively) using CL or EG for 7.77 ± 4.77 years were included in the study. After a brief anamnesis, a simple examination of the ocular surface was performed without staining. All CL group subjects used soft CLs (36–67% of water) at least 8 h daily. In total, 25% of the women took oral contraceptives (33.3% EG and 17.3% CL). The research followed the tenets of the Declaration of Helsinki and the subjects signed an informed consent form to a protocol approved by the Ethics Committee of the University Miguel Hernandez. Table 1 summarizes the characteristics of the participants.

### 2.2. OSDI Questionnaire

All the volunteers completed a self-administered Spanish version of the OSDI questionnaire [10]. The values given by the volunteers to each question, the sum of the values scored to the questions of each subscale (OSDI A: ocular symptoms; OSDI B: vision-related functionality; OSDI C: environmental factors acting as triggers) and the total OSDI score (OSDI D) were recorded. Volunteers with an OSDI D score ≥ 13 were classified as symptomatic as previously reported in the literature [1,27].

### 2.3. Tear Volume and TBUT

In order to study the relationship between tear volume and TBUT values and the OSDI scores in young EG and CL wearers, tear volume (measured without topical anesthesia using a phenol red thread -Zone-Quick; Menicon, Tokyo, Japan- carefully placed during 15 s in the inferior conjunctival sac, near the temporal canthus, and defined as the millimeters of thread moistened during that time) and TBUT (measured under slit lamp examination; fluorescein was applied with a commercial fluorescein strip -Optitech Eyecare, Prayagraj, India- wetted with saline solution) were measured in 47 of the 134 volunteers (calculated sample size *n* = 20) while not wearing CL or EG. Values obtained in both eyes of each subject were averaged and considered as a single datum.

### 2.4. Data Analysis

The statistical analysis was carried out using SPPS v.25 software (IBM, Armonk, NY, USA). Data were expressed as mean ± SEM and were compared using parametric or their equivalent nonparametric test, as indicated. Correlation between OSDI, tear volume and TBUT was also calculated.

Logistic regression and confusion matrix were performed to evaluate the accuracy of the symptomatic/asymptomatic classification using Excel 2010 (Microsoft Corporation, Redmond, WA, USA) and Google Colab (Google LLC, Santa Clara, CA, USA) with Jupyter Notebooks, libraries scikit-learn 0.22.2.post1, Pandas v0.25.3 and Matplotlib Python v3.2.0. Logistic regression is a quite simple and effective algorithm of classification used to define the best-fitting model describing the relationship between two variables. For the logistic regression, each OSDI question was considered as 1 when the score for the question was ≥1, and 0 when the score of the questions was 0. Statistical significance of logistic regression coefficients was determined using the test of conformity. Confusion matrix is a very intuitive and easy metric used to find the accuracy of a classification model, and was used to compare the symptomatic/asymptomatic classifications we performed based in the OSDI D scores (>13), the logistic regression model and the clinical signs (reduced tearing and/or TBUT). The confusion matrix C (count of true negatives was C_0,0_, false negatives was C_1,0_, true positives was C_1,1_ and false positives was C_0,1_) was calculated first for the OSDI classification (true label) compared with the calculated logistic regression model (predicted label). Then, it was calculated for the OSDI classification (true label) and the asymptomatic/symptomatic classification based on clinical signs (predicted label).

The significance level was set as *p* < 0.05 in all statistical analyses.

## 3. Results

### 3.1. OSDI Questions on Ocular Discomfort (OSDI A) Were Not Scored Significantly Higher by CL Symptomatic Subjects

Mean scores for the A, B and C subscales and OSDI D (total OSDI punctuation) were significantly higher in CL wearers than in EG wearers (Table 2), suggesting that CL wearers were closer to developing DED than EG wearers. After the classification as asymptomatic or symptomatic subjects using the OSDI D score, a significantly higher proportion of symptomatic subjects was found in the CL group (Table 2). From the women taking oral contraceptives, only one (a CL wearer) was classified as symptomatic with an OSDI D = 13.

When comparing asymptomatic versus symptomatic EG wearers, values given to each OSDI question were significantly higher in the symptomatic than in the asymptomatic subjects (Table 3). In CL wearers, only the values given to part of the OSDI questions were higher in symptomatic than in asymptomatic subjects, particularly in those about performing activities (OSDI B) and environmental factors (OSDI C) (Table 3). Asymptomatic CL wearers always rated “none of the time” or “some of the time” in the functionality questions, so none of them scored equal or higher than “half of the time”. Surprisingly, scores of OSDI A questions on ocular discomfort were not significantly different in asymptomatic and symptomatic CL wearers (Table 3).

### 3.2. CL Wearers Were Classified as Symptomatic Mainly by Their Score of OSDI Questions on Functionality (OSDI B)

**Figure 1:**
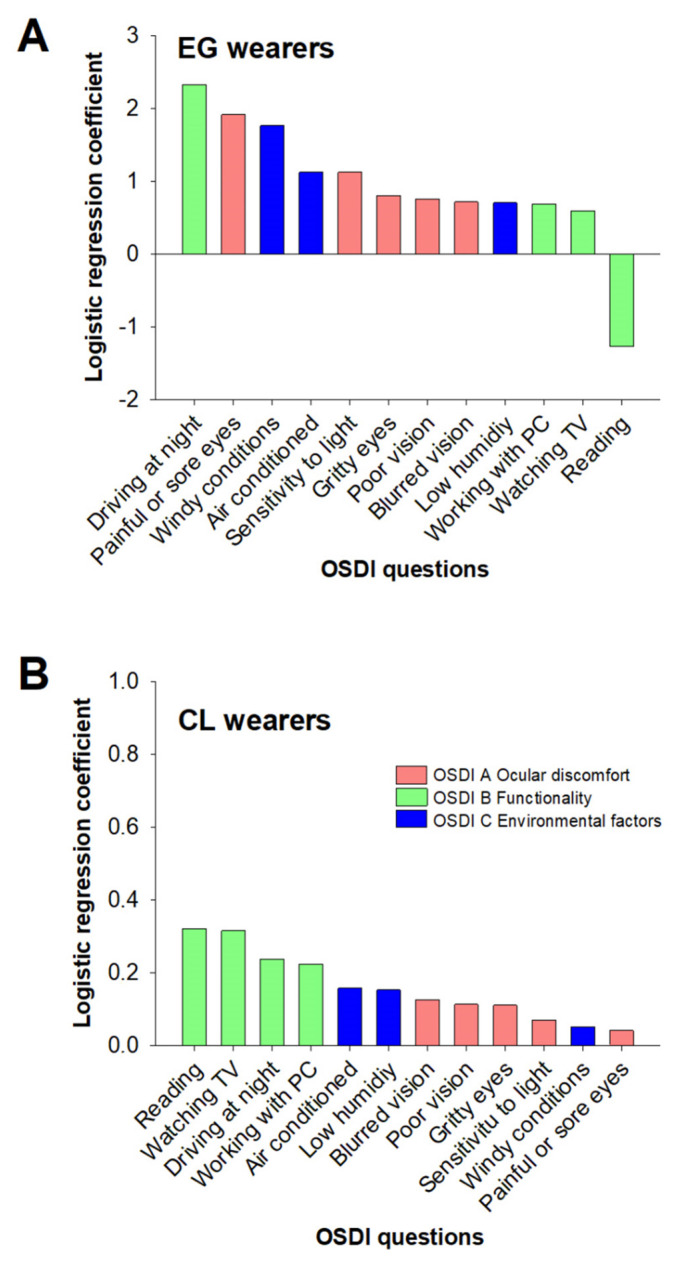
Logistic regression coefficients calculated for the twelve OSDI questions in young eyeglasses (EG) (**A**) and contact lens (CL) (**B**) wearers. In the EG group, the values given to all the questions contributed significantly (*p* < 0.001, *t*- test of conformity). In the CL group, only the score given to “reading” was nearly significant (*p* = 0.05, *t*-test of conformity).

To find out which OSDI questions were determinant to classifying one subject as symptomatic, a logistic regression analysis was performed for both groups, the CL and EG wearers. In the EG group, the values given to all the questions contributed significantly (*p* < 0.001, *t*- test of conformity), with highest logistic regression coefficients obtained for the questions “driving at night”, “painful or sore eyes”, “windy conditions”, “areas that are air conditioned” and “sensitivity to light” (Figure 1A), belonging to all three subscales (OSDI A, OSDI B and OSDI C).

For CL wearers, logistic regression coefficients lower than those of EG wearers were obtained. Noteworthy, the highest logistic regression coefficients obtained in the CL group were for the questions on “reading”, “driving at night”, “working with PC” and “watching tv” (Figure 1B), all four questions being related to functionality (OSDI B) and being nearly significant for the classification as symptomatic, subject only to the score given to “reading” (*p* = 0.05, *t*-test of conformity).

**Figure 2:**
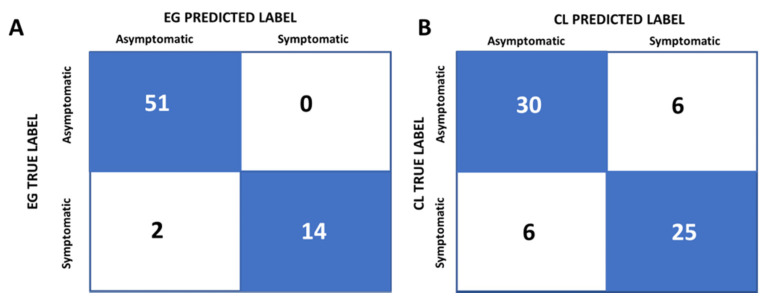
Confusion matrix for the classification of young eyeglasses (EG) (**A**) and contact lens (CL) (**B**) wearers as asymptomatic or symptomatic using OSDI D scores (true label) and logistic regression prediction (predicted label). The number of subjects was shown inside each box.

The confusion matrix C was then calculated for the asymptomatic/symptomatic classification according to the OSDI values (true label) and the logistic regression model (predicted label). For the EG group, the confusion matrix showed that 97% of the subjects were “correctly” classified (76.1% asymptomatic; 20.9% symptomatic) (Figure 2A, blue boxes), while the remaining 3% symptomatic subjects were “incorrectly” classified as asymptomatic (Figure 2A, white boxes). For the CL group, 82.1% were “correctly” classified (44.8% asymptomatic; 37.5% symptomatic) (Figure 2B, blue boxes) and 17.9% of the subjects were “incorrectly” classified (half of them as symptomatic and the other half as asymptomatic) (Figure 2B, white boxes). Overall, OSDI misclassified more CL than EG subjects as symptomatic/asymptomatic.

### 3.3. OSDI D Classified More Symptomatic CL Wearers Than Clinical Eye Dryness Signs

Tear volume and TBUT were measured in 47 of the 134 subjects as clinical signs of DED. Subjects were classified as symptomatic depending on both their OSDI-D score (≥13) and the presence of DED signs (tearing < 10 mm and/or a TBUT < 7 s) (Table 4). Although similar percentages of symptomatic and asymptomatic subjects were found using OSDI and DED clinical signs (Table 4), some subjects were differently classified with both methods. Only 2 of 24 EG users were classified as symptomatic based on their clinical signs. Additionally, no correlation was found between tear and TBUT values and OSDI scores (Pearson correlation analysis, *p* > 0.05).

The 43.5% of CL users were classified as symptomatic based on OSDI and only 39.1% based on clinical signs (Table 4), most of them because they had short TBUT, although the tear volume was normal. Despite most values being over 10 mm, the tear volume values of CL wearers were negatively correlated with OSID B, C and D scores (Pearson correlation coefficients: −0.415, −0.447 and −0.434 for OSDI B, C and D, respectively; *p* < 0.05), supporting the idea that even small decreases in tear volume (below clinical significance) were important in terms of inducing the subjective symptoms measured by OSDI.

**Figure 3:**
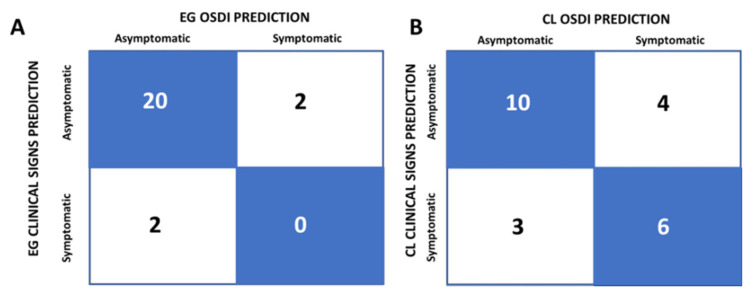
Confusion matrix for the classification of young eyeglasses (EG) (**A**) and contact lens (CL) (**B**) wearers as asymptomatic or symptomatic using OSDI D scores and the presence of clinical signs (reduced tear rate and/or TBUT). The number of subjects was shown inside each box.

Next, the confusion matrix was calculated to compare the classification as symptomatic/asymptomatic according to OSDI and DED clinical signs. The 83.3% of EG wearers were “correctly” classified as asymptomatic by OSDI (Figure 3A, blue boxes), while 16.6% of EG wearers were “incorrectly” classified (8.3% as symptomatic and 8.3% as asymptomatic) (Figure 3A, white boxes). In CL wearers, the percentage of subjects “correctly” classified was 69.6% (43.5% classified as asymptomatic and 26.1% as symptomatic) (Figure 3B, blue boxes), while the remaining 30.1% were “incorrectly” classified (13% as asymptomatic and 17.4% as symptomatic) (Figure 3B, white boxes). Again, OSDI misclassified more subjects from the CL group than from the EG group, although it was more precise than the development of clinical signs to detecting or classifying symptomatic subjects.

## 4. Discussion

In the present work, we used the OSDI questionnaire to classify symptomatic and asymptomatic young EG and CL wearers, identifying if all twelve questionnaire items contributed equally or not to the classification. Only young individuals with no DED history were included in the study, so that even those classified as symptomatic subjects in this study were in preclinical stages of DED.

The present results showed that the relevance of questions belonging to the different OSDI subscales was not exactly the same for EG and CL wearers to achieve the symptomatic level of 13 points. All the questions of OSDI A, B and C (related to ocular discomfort, functionality and environmental factors, respectively) appeared to contribute similarly to identify symptomatic EG wearers. Although both the OSDI B and OSDI C question were scored significantly higher values by symptomatic CL wearers, the logistic regression showed that the OSDI B questions related to “functionality”, that is the difficulty to perform vision-related daily life activities (denoting quality of life), were more relevant to classify symptomatic CL wearers. Surprisingly, symptomatic CL subjects did not give high scores to the questions on “ocular discomfort” (OSDI A), showing that ocular discomfort was not a main symptom in these young CL wearers and supporting the idea that the symptomatic CL wearers should be at the preclinical or early DED stage.

It is very important that dry eye and discomfort questionnaires include items evaluating the impact of ocular dryness on daily life activities (which are directly related to the quality of life [36], because those items can bring to light indirect symptoms in early DED stages, as some authors have suggested [21,32,33,35]. Although the use of more recent questionnaires, such as the standard patient evaluation of eye dryness (SPEED) [8,39], the symptom assessment in dry eye (SANDE) [7,40] or the subjective evaluation of symptom of dryness (SESoD) [37], also identifies symptomatic subjects, the TFOS DEWS II report recommends the OSDI questionnaire for evaluating DED symptoms [27]. The present confusion matrices analysis also supported this idea, as OSDI D had a strong discrimination power between symptomatic subjects [1,41], with a high specificity and sensibility in young EG and CL wearers, although in a slightly lesser degree in the latter group. The OSDI questionnaire gave more symptomatic false positives and false negatives in the CL group than in the EG group. The CL composition and the number of hours they were worn per day could influence corneal sensitivity and, hence, OSDI values [42]. In our study population, all of the CL wearers used quite similar soft CLs (with a high water content) and wore them daily for similar times, so that the difference in OSDI D leading to their classification as symptomatic or asymptomatic probably could not be attributable to these variables.

Questionnaires that do not consider the effect on daily life activities are not expected to have the same accuracy to detect symptomatic subjects. That would be the case of the simple SESoD questionnaire, which has just one question to quantify ocular dryness along the day [37]. Its use has increased (probably due to its speed of use) to distinguish between symptomatic and asymptomatic subjects in DED patients, but also to discriminate between symptomatic and asymptomatic CL wearers [25,37,43,44,45,46]. Despite its simplicity, some studies have reported its positive correlation with other questionnaires, such as the dry eye questionnaire (DEQ) [47], the McMonnies dry eye questionnaire (MQ) [37], the SANDE [40] or SPEED questionnaires [39]. Indeed, the SESoD questionnaire has been validated to detect symptomatic subjects in a comparable way to OSDI, showing that both questionnaires give similar results to detecting symptomatic subjects, with a positive correlation between SESoD and OSDI scores [37]. However, that study did not check the efficacy of both tests to classify each subject.

We also studied the relationship between the tear volume and TBUT values and the OSDI scores in young EG and CL wearers. Even though the sample size of these measurements was smaller than for the OSDI measurements, the results were strong enough to observe that OSDI could detect more symptomatic subjects than the clinical measurements, as previously observed [13]. Only 8% of the EG young subjects presented clinical signs, and no correlation was found between their tear rate or TBUT values and OSDI scores. This was not the case for CL wearers, whose tear volume values were normal but correlated significantly with the OSDI scores. This suggested that even very small reductions in tearing may be a sign (and/or the cause) of the pathological state producing dry eye symptoms in young CL wearers, who would probably develop DED later. Although a reduced tearing rate and TBUT are commonly used as clinical signs to diagnose DED, they are not always reduced in OSDI-detected symptomatic subjects, and vice versa [9,10,12,13,48]. The confusion matrix performed to analyze the symptomatic/asymptomatic classification with the OSDI scores and the clinical sign values showed that more CL wearers were classified as symptomatic by OSDI than by the presence of clinical signs, suggesting that the OSDI questionnaire is more useful to diagnose symptomatic subjects with mild or preclinical DED whose tear deficit and/or instability is not enough to be clinically detected. This could be the case for the young CL wearers that we classified as symptomatic due to their OSDI D scores being approximately 13. The OSDI questionnaire was a very useful tool to identify those individuals who still did not present signs or symptoms, probably because they were currently at a subclinical early state, but would potentially develop DED. It has been previously reported that some questionnaires may detect early symptoms in subjects without clear clinical signs [13], this being the main reason as to why the questionnaire scores sometimes did not correlate with the clinical signs.

Although it is generally assumed that OSDI can detect DED, it cannot be excluded that, in some cases (as could be the case for young CL wearers with OSDI scores of approximately 13), OSDI could probably just be detecting “ocular surface dryness/discomfort” without DED itself. Along the same lines, the most frequent symptoms in CL users are dryness and discomfort [20,21], and the use of CL is a clear risk of developing DED [26,27]. However, the present results showed that young CL wearers did not score high values on the OSDI questions on ocular discomfort (OSDI A).

Taken together, results showed that the OSDI questionnaire was useful in identifying young CL users with no clinical signs of DED, but at risk of developing symptoms. Young contact lens wearers with an OSDI D ≥ 13 and scoring 2 or higher on the OSDI questions on vision-related daily life activities should be considered symptomatic patients and subjected to a further clinical evaluation, helping to start preventive treatments before DED is fully established.

## Figures and Tables

**Table 1:** Characteristics of the eyeglasses (EG) wearers and soft contact lens (CL) wearers participating in the study.

	EG	CL
Age (years)	23.19 ± 0.42	23.18 ± 0.53
Gender ratio (men:women)	16:51	15:52
Corrective lenses wearing history (years)	7.49 ± 0.66	8.06 ± 0.5
*n*	67	67

Data are mean ± s.e.m.; *n*—number of subjects. No differences between EG and CL groups, *t*-test and *z*-test.

**Table 2:** OSDI subscale scores and total OSDI score in eye glasses (EG) wearers and soft contact lens (CL) wearers.

OSDI	EG (*n* = 67)	CL (*n* = 67)
OSDI A (Ocular symptoms)	4.09 ± 0.38	4.91 ± 0.3 *
OSDI B (Functionality)	2.51 ± 0.43	3.12 ± 0.40 *
OSDI C (Environmental factors)	2.55 ± 0.33	4.66 ± 0.39 *
OSDI D (Total)	9.15 ± 0.93	12.60 ± 0.90 **
Symptomatic subjects (OSDI D ≥ 13)	23.9%	46.3% ^†^

Data are mean ± s.e.m. *n*—number of subjects. Differences between EG and CL wearers: ** *p* < 0.001, * *p* < 0.01, *t*-test; ^†^ *p* < 0.05, *z*-test.

**Table 3:** Scores of OSDI questions obtained in asymptomatic (A) and symptomatic (S) eyeglasses (EG) and contact lens (CL) wearers.

	EG		CL	
	A (*n* = 51)	S (*n* = 16)	A (*n* = 36)	S (*n* = 31)
**Have you experienced any of the following during the last week?**				
Sensitivity to light?	1.2 ± 0.14 (31.4)	2.69 ± 0.31 * (75)	1.33 ± 0.18 (33.3)	2.03 ± 0.22 (58.1)
2. Gritty eyes?	0.16 ± 0.05 (0)	0.81 ± 0.28 ** (25)	0.31± 0.09 (2.8)	0.68 ± 0.12 (12.9)
3. Painful or sore eyes?	0.49 ± 0.09 (5.9)	1.44 ± 0.24 * (37.5)	0.69 ± 0.09 (2.8)	1.16 ± 0.15 ** (29)
4. Blurred vision?	0.59 ± 0.11 (9.8)	1.38 ± 0.24 ^†^ (37.5)	0.75 ± 0.13 (5.6)	1.26 ± 0.15 (22.6)
5. Poor vision?	0.49 ± 0.09 (3.9)	1.63 ± 0.3 * (43.8)	0.58 ± 0.14 (8.3)	1.10 ± 0.18 (22.6)
**Have problems with your eyes limited you in performing any of the following during the last week?**				
6. Reading?	0.27 ± 0.08 (2)	1.06 ± 0.39 * (25)	0.14 ± 0.06 (0)	1.39 ± 0.2 ** (38.7)
7. Driving at night?	0.55 ± 0.2 (3.9)	3.06 ± 0.39 ** (56.25)	0.83 ± 0.29 (0)	2.58 ± 0.38 ** (19.35)
8. Working with a computer?	0.47 ± 0.09 (15.6)	2.19 ± 0.38 ** (56.3)	0.36 ± 0.08 (0)	1.77 ± 0.2 ** (58.1)
9. Watching TV?	0.39 ± 0.12 (2)	1.88 ± 0.36 ** (50)	0.19 ± 0.07 (0)	1.48 ± 0.18 ** (45.2)
**Have your eyes felt uncomfortable in any of the following situations during the last week?**				
10. Windy conditions?	0.69 ± 0.11 (11.8)	2.31 ± 0.31 ** (20.8)	1.22 ± 0.15 (27.8)	2.58 ± 0.19 ** (83.9)
11. Places or areas with low humidity (very dry)?	0.31 ± 0.86 (7.8)	1.5 ± 0.33 ** (50)	0.67 ± 0.16 (5.6)	1.77 ± 0.24 ** (51.6)
12. Areas that are air conditioned?	0.59 ± 0.12 (13.7)	1.75 ± 0.31 ** (56.3)	1 ± 0.2 (22.2)	2.16 ± 0.23 ** (67.7)

Volunteers were classified as symptomatic when their total OSDI score was ≥ 13. Data are mean ± s.e.m.; *n*—number of subjects. Percentages of subjects with a positive response (equal or higher than “half of the time”) to each question are also shown in brackets. Comparisons between EG-S and CL-S found no statistical differences; ** *p* < 0.001, * *p* < 0.01; ^†^ *p* < 0.05, *t*-test or the Mann–Whitney U test, differences between A and S subjects.

**Table 4:** Classification of EG and CL wearers as symptomatic subjects depending on the OSDI total score values or the presence of clinical dry eye signs (reduced tear volume and/or TBUT).

		EG (*n* = 24;15 W/9 M)	CL (*n* = 23;17 W/6 M)
OSDI	OSDI D	6.0 ± 1.0	12.6 ± 1.2 **
	Symptomatic subjects ^1^	2 (8.3%)	10 (43.5% ^†^)
Signs	Tear volume (mm)	26.8 ± 1.6	25.2 ± 2.0
	TBUT (s)	11.3 ± 0.5	8.2 ± 0.5 **
	Symptomatic subjects ^2^	2 (8.3%)	9 (39.1% ^†^)

Data are mean ± s.e.m.; *n*—number of subjects. Number of women (W) and men (M) was also indicated. Differences between EG and CL wearers: ** *p* < 0.001, *t*-test or Mann–Whitney; ^†^ *p* < 0.05, *z*-test. ^1^ Criterion to be classified as symptomatic, OSDI total score ≥ 13; ^2^ Criterion to be classified as symptomatic, tear volume < 10 mm and/or TBUT < 7 s.

## Data Availability

The original contributions presented in the study are included in the article. Further inquiries can be directed to the corresponding author.

## References

[1] Schiffman R.M., Christianson M.D., Jacobsen G., Hirsch J.D., Reis B.L. (2000). Reliability and validity of the ocular surface disease index. Arch. Ophthalmol..

[2] Chalmers R.L., Begley C.G., Caffery B. (2010). Validation of the 5-Item Dry Eye Questionnaire (DEQ-5): Discrimination across self-assessed severity and aqueous tear deficient dry eye diagnoses. Contact Lens Anterior Eye.

[3] Sakane Y., Yamaguchi M., Yokoi N., Uchino M., Dogru M., Oishi T., Ohashi Y., Ohashi Y. (2013). Development and validation of the dry eye-related quality-of-life score questionnaire. JAMA Ophthalmol..

[4] Abetz L., Rajagopalan K., Mertzanis P., Begley C., Barnes R., Chalmers R. (2011). Development and validation of the impact of dry eye on everyday life (IDEEL) questionnaire, a patient-reported outcomes (PRO) measure for the assessment of the burden of dry eye on patients. Health Qual. Life Outcomes.

[5] McMonnies C.W., Ho A. (1987). Responses to a dry eye questionnaire from a normal population. J. Am. Optom. Assoc..

[6] Johnson M.E., Murphy P.J. (2007). Measurement of ocular surface irritation on a linear interval scale with the ocular comfort index. Investig. Ophthalmol. Vis. Sci..

[7] Schaumberg D.A., Gulati A., Mathers W.D., Clinch T., Lemp M.A., Nelson J.D., Foulks G.N., Dana R. (2007). Development and validation of a short global dry eye symptom index. Ocul. Surf..

[8] Blackie C.A., Solomon J.D., Scaffidi R.C., Greiner J.V., Lemp M.A., Korb D.R. (2009). The relationship between dry eye symptoms and lipid layer thickness. Cornea.

[9] Begley C.G., Caffery B., Chalmers R.L., Mitchell G.L. (2002). Use of the dry eye questionnaire to measure symptoms of ocular irritation in patients with aqueous tear deficient dry eye. Cornea.

[10] Fuentes-Páez G., Herreras J.M., Cordero Y., Almaraz A., González M.J., Calonge M. (2011). Lack of concordance between dry eye syndrome questionnaires and diagnostic tests. Arch. Soc. Española Oftalmol..

[11] Pult H., Purslow C., Murphy P.J. (2011). The relationship between clinical signs and dry eye symptoms. Eye.

[12] Ünlü C., Güney E., Akçay B.I.S., Akçali G., Erdoǧan G., Bayramlar H. (2012). Comparison of ocular-surface disease index questionnaire, tearfilm break-up time, and Schirmer tests for the evaluation of the tearfilm in computer users with and without dry-eye symptomatology. Clin. Ophthalmol..

[13] McMonnies C.W. (2021). Why the symptoms and objective signs of dry eye disease may not correlate. J. Optom..

[14] Nichols J.J., Willcox M.D.P., Bron A.J., Belmonte C., Ciolino J.B., Craig J.P., Dogru M., Foulks G.N., Jones L., Nelson J.D. (2013). The TFOS International Workshop on Contact Lens Discomfort: Executive summary. Investig. Ophthalmol. Vis. Sci..

[15] Chalmers R.L., Keay L., McNally J., Kern J. (2012). Multicenter case-control study of the role of lens materials and care products on the development of corneal infiltrates. Optom. Vis. Sci..

[16] Paquette L., Jones D.A., Sears M., Nandakumar K., Woods C.A. (2015). Contact lens fitting and training in a child and youth population. Contact Lens Anterior Eye.

[17] Stapleton F., Keay L., Edwards K., Naduvilath T., Dart J.K.G., Brian G., Holden B.A. (2008). The Incidence of Contact Lens-Related Microbial Keratitis in Australia. Ophthalmology.

[18] Chalmers R.L., Keay L., Long B., Bergenske P., Giles T., Bullimore M.A. (2010). Risk factors for contact lens complications in US clinical practices. Optom. Vis. Sci..

[19] Chalmers R.L., Wagner H., Lynn Mitchell G., Lam D.Y., Kinoshita B.T., Jansen M.E., Richdale K., Sorbara L., McMahon T.T. (2011). Age and other risk factors for corneal infiltrative and inflammatory events in young soft contact lens wearers from the Contact Lens Assessment in Youth (CLAY) study. Investig. Ophthalmol. Vis. Sci..

[20] Muntz A., Subbaraman L.N., Sorbara L., Jones L. (2015). Tear exchange and contact lenses: A review. J. Optom..

[21] Stapleton F., Alves M., Bunya V.Y., Jalbert I., Lekhanont K., Malet F., Na K.S., Schaumberg D., Uchino M., Vehof J. (2017). TFOS DEWS II Epidemiology Report. Ocul. Surf..

[22] Dumbleton K., Woods C.A., Jones L.W., Fonn D. (2013). The impact of contemporary contact lenses on contact lens discontinuation. Eye Contact Lens..

[23] Riley C., Young G., Chalmers R. (2006). Prevalence of ocular surface symptoms, signs, and uncomfortable hours of wear in contact lens wearers: The effect of refitting with daily-wear silicone hydrogel lenses (senofilcon A). Eye Contact Lens..

[24] Martín-Montañez V., López-de la Rosa A., López-Miguel A., Pinto-Fraga J., González-Méijome J.M., González-García M.J. (2015). End-of-day dryness, corneal sensitivity and blink rate in contact lens wearers. Contact Lens Anterior Eye.

[25] Woods C.A., Bentley S.A., Fonn D. (2016). Temporal changes in contact lens comfort over a day of wear. Ophthalmic Physiol. Opt..

[26] Craig J.P., Nichols K.K., Akpek E.K., Caffery B., Dua H.S., Joo C.K., Liu Z., Nelson J.D., Nichols J.J., Tsubota K. (2017). TFOS DEWS II Definition and Classification Report. Ocul. Surf..

[27] Wolffsohn J.S., Arita R., Chalmers R., Djalilian A., Dogru M., Dumbleton K., Gupta P.K., Karpecki P., Lazreg S., Pult H. (2017). TFOS DEWS II Diagnostic Methodology report. Ocul. Surf..

[28] Begley C.G., Chalmers R.L., Mitchell G.L., Nichols K.K., Caffery B., Simpson T., DuToit R., Portello J., Davis L. (2001). Characterization of ocular surface symptoms from optometric practices in North America. Cornea.

[29] Chalmers R.L., Begley C.G., Moody K., Hickson-Curran S.B. (2012). Contact Lens Dry Eye Questionnaire-8 (CLDEQ-8) and opinion of contact lens performance. Optom. Vis. Sci..

[30] Pesudovs K., Garamendi E., Elliott D.B. (2006). A quality of life comparison of people wearing spectacles or contact lenses or having undergone refractive surgery. J. Refract. Surg..

[31] Arroyo-Del Arroyo C., Fernández I., López-de la Rosa A., Pinto-Fraga J., González-García M.J., López-Miguel A. (2022). Design of a questionnaire for detecting contact lens discomfort: The Contact Lens Discomfort Index. Clin. Exp. Optom..

[32] Patel V.D., Watanabe J.H., Strauss J.A., Dubey A.T. (2011). Work productivity loss in patients with dry eye disease: An online survey. Curr. Med. Res. Opin..

[33] Yamada M., Mizuno Y., Shigeyasu C. (2012). Impact of dry eye on work productivity. Clin. Outcomes Res..

[34] Grubbs J.R., Tolleson-Rinehart S., Huynh K., Davis R.M. (2014). A Review of Quality of Life Measures in Dry Eye Questionnaires. Cornea.

[35] Nichols K.K., Bacharach J., Holland E., Kislan T., Shettle L., Lunacsek O., Lennert B., Burk C., Patel V. (2016). Impact of dry eye disease on work productivity, and patients’ Satisfaction with Over-The-Counter dry eye treatments. Investig. Ophthalmol. Vis. Sci..

[36] Sayegh R.R., Yu Y., Farrar J.T., Kuklinski E.J., Shtein R.M., Asbell P.A., Maguire M.G. (2021). Ocular Discomfort and Quality of Life Among Patients in the Dry Eye Assessment and Management Study. Cornea.

[37] Simpson T.L., Situ P., Jones L.W., Fonn D. (2008). Dry eye symptoms assessed by four questionnaires. Optom. Vis. Sci..

[38] Saldanha I.J., Petris R., Han G., Dickersin K., Akpek E.K. (2018). Research Questions and Outcomes Prioritized by Patients with Dry Eye. JAMA Ophthalmol..

[39] Asiedu K., Kyei S., Mensah S.N., Ocansey S., Abu L.S., Kyere E.A. (2016). Ocular surface disease index (OSDI) versus the standard patient evaluation of eye dryness (SPEED): A study of a nonclinical sample. Cornea.

[40] Amparo F., Schaumberg D.A., Dana R. (2015). Comparison of Two Questionnaires for Dry Eye Symptom Assessment: The Ocular Surface Disease Index and the Symptom Assessment in Dry Eye. Ophthalmology.

[41] Pult H., Murphy P.J., Purslow C. (2009). A novel method to predict the dry eye symptoms in new contact lens wearers. Optom. Vis. Sci..

[42] García-Porta N., Rico-del-Viejo L., Martin-Gil A., Carracedo G., Pintor J., González-Méijome J.M. (2016). Differences in Dry Eye Questionnaire Symptoms in Two Different Modalities of Contact Lens Wear: Silicone-Hydrogel in Daily Wear Basis and Overnight Orthokeratology. BioMed Res. Int..

[43] Srinivasan S., Chan C., Jones L. (2007). Apparent time-dependent differences in inferior tear meniscus height in human subjects with mild dry eye symptoms. Clin. Exp. Optom..

[44] Hom M.M., Bruce A.S. (2009). Prelens tear stability: Relationship to symptoms of dryness. Optometry.

[45] Chen J., Simpson T.L. (2011). A role of corneal mechanical adaptation in contact lens-related dry eye symptoms. Investig. Ophthalmol. Vis. Sci..

[46] Wang K., Tobillo R., Mavroidis P., Pappafotis R., Pearlstein K.A., Moon D.H., Mahbooba Z.M., Deal A.M., Holmes J.A., Sheets N.C. (2019). Prospective Assessment of Patient-Reported Dry Eye Syndrome After Whole Brain Radiation. Int. J. Radiat. Oncol. Biol. Phys..

[47] Okumura Y., Inomata T., Iwata N., Sung J., Fujimoto K., Fujio K., Midorikawa-Inomata A., Miura M., Akasaki Y., Murakami A. (2020). A review of dry eye questionnaires: Measuring patient-reported outcomes and health-related quality of life. Diagnostics.

[48] Muselier-Mathieu A., Bron A.M., Mathieu B., Souchier M., Brignole-Baudouin F., Acar N., Brétillon L., Creuzot-Garcher C. (2014). Ocular surface assessment in soft contact lens wearers; The contribution of tear osmolarity among other tests. Acta Ophthalmol..

